# A qualitative assessment of internal medicine resident perceptions of graduate medical education following implementation of the 2011 ACGME duty hour standards

**DOI:** 10.1186/1472-6920-14-84

**Published:** 2014-04-22

**Authors:** Christa R Nevin, Andrea Cherrington, Brita Roy, David D Daly, J Martin Rodriguez, Mukesh Patel, Erin D Snyder, Angelo L Gaffo, Joseph Barney, James H Willig

**Affiliations:** 1 University of Alabama at Birmingham, 845 19th Street South, BBRB 220B, Birmingham, AL 35294, USA; 2 Robert Wood Johnson Foundation Clinical Scholars Program with support from the US Veterans Administration, Yale University, New Haven, CT, USA; 3 Medical University of South Carolina, Charleston, SC, USA; 4 University of Maryland Baltimore, Baltimore, MD, USA

**Keywords:** Medical education, Medical education-graduate, Qualitative research

## Abstract

**Background:**

In 2011, the Accreditation Council of Graduate Medical Education implemented updated guidelines for medical resident duty hours, further limiting continuous work hours for first-year residents. We sought to investigate the impact of these restrictions on graduate medical education among internal medicine residents.

**Methods:**

We conducted eight focus groups with internal medicine residents at the University of Alabama at Birmingham in 06/2012-07/2012. Discussion questions included, “*How do you feel the 2011 ACGME work hour restrictions have impacted your graduate medical education?*” Transcripts of the focus groups were reviewed and themes identified using a deductive/inductive approach. Participants completed a survey to collect demographic information and future practice plans.

**Results:**

Thirty-four residents participated in our focus groups. Five themes emerged: decreased teaching, decreased experiential learning, shift-work mentality, tension between residency classes, and benefits and opportunities. Residents reported that since implementation of the guidelines, teaching was often deferred to complete patient-care tasks. Residents voiced concern that PGY-1 s were not receiving adequate clinical experience and that procedural and clinical reasoning skills are being negatively impacted. PGY-1 s reported being well-rested and having increased time for independent study.

**Conclusions:**

Residents noted a decline in teaching and are concerned with the decrease in “hands-on” clinical education that is inevitably impacted by fewer hours in the hospital, though some benefits were also reported. Future studies are needed to further elucidate the impact of decreased resident work hours on graduate medical education.

## Background

In July 2003, the Accreditation Council for Graduate Medical Education (ACGME) mandated a reduction in duty hours for medical residents in accredited programs. These guidelines limited residents of all levels to no more than 30-hours of continuous work and to an 80-hour work week. This was done primarily to reduce the number of errors attributed to resident fatigue and to improve resident quality of life [1]. The impact of the 2003 ACGME duty hour standards on graduate medical education is not fully understood, and study results thus far have been mixed. While some studies indicate that few, if any, educational outcomes have been impacted by the decrease in duty hours, others indicate that residents have fewer clinical experiences, lower scores on standardized tests, fewer publications and less participation in academic medicine, and decreases in attending physician teaching and other educational opportunities [2-17].

Additionally, in response to recommendations by the Institute of Medicine (IOM), the ACGME further revised duty hour guidelines in July 2011 that limited continuous work hours for PGY-1 medical residents to no more than 16-hours and upper level residents to 28-hours [1]. Little is known about the impact of the 2011 ACGME duty hour standards on resident education. The controversy surrounding educational outcomes from the 2003 guidelines, in addition to further restrictions in the 2011 guidelines, merits an in-depth understanding of how work-hour changes have specifically impacted resident learning. In this study, we assess perceptions among internal medicine residents regarding the impact of the 2011 ACGME duty hour standards on graduate medical education.

## Methods

### Setting

The University of Alabama at Birmingham (UAB) Internal Medicine Residency program is a large training program affiliated with an academic medical center in Birmingham, Alabama. During the 2011–2012 academic year, 128 total residents were completing their internal medicine training at UAB. Internal medicine residents at UAB completed their inpatient rotations at three large medical centers: UAB Hospital, a quarternary care academic center, the Birmingham Veteran’s Administration Hospital, and Cooper Green Mercy Hospital, a county hospital. Inpatient rotations occured on general internal medicine and subspecialty wards, including pulmonology, gastroenterology, nephrology, hematology/oncology, cardiology, and intensive care.

Prior to the 2011 duty hour restrictions, general internal medicine ward teams were composed of 1 attending physician, 1 senior resident (PGY 2–3), 2 interns (PGY-1), and 1–2 third or fourth year medical student. Senior residents were responsible for new admissions every fourth or fifth day, completing 12-hour shifts on weekdays and 30-hour shifts on weekends. A night float senior resident worked 5 consecutive weeknights (Sunday-Thursday) with primary responsibility for new admissions from 7 pm-7 am. Interns completed 30-hour shifts every fourth to eighth night and a 30-hour shift on weekends. A separate night float intern took responsibility for cross-coverage from 7 pm-7 am, Sunday-Thursday.

After the implementation of 2011 AGME duty hour restrictions, the composition of general medicine ward teams and night float coverage did not change. The schedule for senior residents remained the same, albeit limiting overnight shifts to 28 hours. Interns alternated day (7 am-7 pm) and night shifts (7 pm-7 am) for new admissions every fourth or fifth day.

### Study design

We designed a qualitative study to assess perceptions of internal medicine residents regarding the 2011 ACGME duty hours. We conducted a series of eight focus groups with internal medicine residents at UAB between 06/2012-07/2012. The Institutional Review Board at UAB approved this study and all participants provided verbal consent for participation.

### Study recruitment

Investigators invited all postgraduate year one (PGY-1), PGY-2, and PGY-3 internal medicine residents to participate in focus group discussions via recruitment emails and verbal announcements at program conferences. Groups were divided by residency year and no more than eight residents were allowed to participate per group. Participants received a small financial incentive ($35 gift card) for participating.

### Focus groups

Discussions were guided by a series of open-ended questions outlined in a moderator’s (CRN) guide including, “*How do you feel the 2011 ACGME work hour restrictions have impacted your graduate medical education?*” (List 1). In addition, focus group participants were asked to complete a brief survey to collect basic demographic information and future medical practice plans.

List 1: Moderator questions used to guide focus group discussions regarding the impact of the 2011 ACGME duty hour standards on graduate medical education.

1. In what ways and/or what methods do the faculty at UAB employ to teach you while rounding in the hospital wards (e.g. bedside teaching, chalk talks)?

a. [*PROBE*] Which of these methods do you feel is most useful?

b. [*PROBE*] Which of these methods do you feel is least useful?

2. How do you feel that graduate medical education has changed following implementation of the 2011 ACGME work-hour restrictions?

a. [*PROBE*] In what ways are these changes positive?

b. [*PROBE*] In what ways are these changes negative?

3. How do you feel graduate medical education can be improved?

a. [*PROBE*] Using traditional teaching methods?

b. [*PROBE*] Using novel teaching methods?

### Data analysis

Focus groups were audio recorded and transcribed verbatim. Common themes were then coded using a combined deductive and inductive approach until convergence of themes was reached. The transcript of the initial focus group was analyzed first by the principal investigator (CRN) and used to generate preliminary themes that were used as a guide for analysis of remaining transcripts. The transcript was then analyzed by two additional reviewers (JHW and AC) who confirmed preliminary themes. Transcripts from all subsequent focus groups were coded by two independent reviewers (CRN and JHW or AC) and preliminary themes were modified and emerging themes identified and included. Each participant’s response could potentially contribute to more than one identified theme and emerging themes were discussed among the research team prior to inclusion.

## Results

A total of 34 internal medicine residents at UAB participated in eight focus group discussions. The majority of participants (65%) was male and planned to go into subspecialty practice (73%, n = 25). 16 (47%) were PGY-1 residents, 12 (35%) were PGY-2 residents, and six (18%) were PGY-3 residents during the 2011–2012 academic year. Half of participants (50%, n = 73) reported regular study for in-service and board examinations and most (68%, n = 23) stated they spent 0–5 hours per week dedicated solely to preparation for these examinations (Table 1).

**Table 1:** Demographics and study habits of UAB internal medicine residents participating in focus group discussions regarding educational impact of 2011 ACGME duty hour standards (n = 34)

Gender	
Female	12 (35%)
Male	22 (65%)
Residency Training Year	
PGY-1	16 (47%)
PGY-2	12 (35%)
PGY-3	6 (18%)
Future Medical Practice Plans	
Hospitalist	2 (6%)
Primary Care	3 (9%)
Subspecialty	25 (73%)
Undecided	4 (12%)
Regular study for in-service/board examinations	
Yes	17 (50%)
PGY-1	7 (41%)
PGY-2	7 (41%)
PGY-3	3 (18%)
No	15 (44%)
PGY-1	8 (54%)
PGY-2	5 (33%)
PGY-3	2 (13%)
No response	2 (6%)
Hours/week dedicated to studying for examinations	
0-5	23 (68%)
6-10	10 (29%)
11-20	1 (3%)

Five themes emerged following review of the focus group transcripts: 1) decreased teaching, 2) decreased experiential learning, 3) shift-work mentality, 4) tension between resident classes, and 5) benefits and opportunities.

### Decreased teaching

Residents reported that since implementation of the 2011 work-hour restrictions, they felt education was often deferred in order to complete basic patient-care tasks in a timely manner explaining that although the number of work-hours allowed had declined following the ACGME-mandated restrictions, the amount of work to be completed did not decrease. One resident noted:

*You have the same amount of work with less time to put into it. Something’s got to go, and for the most part I think it’s the teaching aspect of things that sort of gets lost in the shuffle.* (PGY-3)

The reduction in time dedicated to education was ascribed to both a decline in teaching by attending physicians, as well as teaching by upper level residents (PGY-2, PGY-3) to interns (PGY-1):

*The amount of time allotted for teaching during morning rounds has become much less now.* (PGY-3)

*It [ACGME work hour restrictions] takes away from the time that third year residents used to spend teaching to the medical students and even teaching to the interns.* (PGY-3)

*They [upper level residents] picked up half our work. It was going to be ours overnight and now it’s theirs, so they are much busier now than they were…So I think it’s a lot harder to find the time than you could have before to teach.* (PGY-1)

Residents affirmed that variations in time dedicated to teaching depended on both the assigned attending physician and service, noting that teaching was affected on general medicine and subspecialty services as well.

### Decreased experiential learning

All residents voiced concern that PGY-1 residents were not receiving an adequate amount of clinical experience and that clinical reasoning and procedural skills were being negatively impacted. Regarding clinical reasoning skills, one PGY-1 resident reported that with the ACGME duty hour standards:

*The thinking process is basically taken out of the equation [because more admissions are done by the night float]. It’s like somebody’s already worked up, thought about it [a patient case], and he sees it a certain way, and that just corrupts the whole process, because now you don’t have to go through [exploring a differential diagnosis].* (PGY-1)

They repeatedly expressed that hands-on clinical experience was their primary learning modality during residency (“*Information seems to be best retained when it can be applied to an individual patient that you’re seeing”)* and that, because of the work hour limitations mandated by the ACGME, opportunity to hone skills in a real world environment under the supervision of senior faculty was being reduced and, in some cases, lost entirely:

*I think one of the really helpful learning experiences for me [comes from] the decisions that I make overnight without direct attending supervision. And then along with that, getting feedback in the mornings from the attendings… And I think that with the work-hour restrictions, they miss out on at least part of those because either they're present during the day or during the night or something, but getting all of that in the same period of time is harder to do with hour restrictions.* (PGY-2)

In addition to an overall reduction of time in the hospital, residents reported decreased opportunity to observe the course and progression of a patient’s disease process and a loss in continuity of patient care (“*My biggest issue with the work hours is the continuity of care gets very broken up”*).

Many upper level residents were concerned that that the new work hour restrictions prevented PGY-1 residents from gaining the clinical experience needed to be fully competent in patient care during their PGY-2 residency year:

I had interns that didn't -- just weren't where they should have been. They were about to be an upper-level resident, and they still did not how to take care of -- really take care of patient in terms of their skill set [and] knowledge.

PGY-1 residents in turn, while acknowledging the decreased opportunity for hands-on experience, reported being confident in their clinical skills moving forward:

I’m ready in the sense that I’m able to do the job…I guess the short answer is … I feel just as ready as somebody else who was an R2 when I was an intern. I don’t feel like I’m any less capable of doing what they were doing at that point.

### Shift-work mentality

All resident classes, including the PGY-1 s, reported that the 2011 ACGME duty hour standards had fostered an environment of “shift-work mentality” and decreased ownership of patients among PGY-1 residents (“*It is shift work and less about ‘This is my patient. I really want them (sic) to get better,’ like less ownership over what you’re doing and more ‘Okay, it’s time for me to go home.”*). Concerns were voiced that such an attitude regarding patient care undermined the educational goals of medical training and the patient safety goals outlined by the ACGME as reasons for the implementation of work-hour restrictions (“*I think you’re seeing more errors. I think the interns are less experienced. There’s (sic) more handoffs. They don’t know their patients. There’s no ownership”*).

### Tension between residency classes

Resentment across the classes and a less positive team dynamic was reported after the implementation of the 2011 ACGME work hour guidelines. Some upper level residents report that these frustrations have impacted their ability to effectively teach PGY-1 residents. From a PGY-2:

When we were interns, I felt like my upper levels respected me and there wasn't any animosity between them or us. And I felt like, you know, there was a better camaraderie, and I find myself resenting a lot of the interns. And I try not to let that impair my willingness to take and teach things and do things with them. But I do definitely feel like it's eroded part of the social contract between the interns and the upper levels.

Conversely, PGY-1 residents reported frustration at the resentment they perceive from the upper level residents and stated that it inhibits their ability to work as a cohesive team:

And really, I think that there is, like, a culture problem that needs to be addressed, because the class above us, in particular, seems to have this very aggrieved… mentality, and it’s tiresome. It really is. And I think it inhibits team cohesiveness, I think it inhibits our ability to be thoughtful members of the team.

### Benefits and opportunities

Despite concerns raised by residents regarding the impact of the 2011 ACGME work hour restrictions on graduate medical education, some positive changes following implementation of the work-hour restrictions were reported. PGY-1 residents reported feeling well-rested and, as a result, more capable of learning:

*There were very few times that I felt sleep deprived and so I felt like it gave me a lot of time to study.* (PGY-1).

In addition, several PGY-1 residents enjoyed the additional time for self-study that the reduced work hours allowed. This depended, however, on personal preference for independent versus group learning:

*I think if you can learn independently by yourself, then the new work hour restrictions…probably actually help you, because you get more independent time. But if you’re like me and you get kind of tired and you lose interest in learning like that and prefer to learn in an environment where you can hear other people’s opinions and then kind of change the topic as you have questions, then the new work hour restrictions kind of, I think, hinder me, especially in that way.* (PGY-1)

Residents also provided several suggestions for how graduate medical education may be improved in the face of the ACGME work hour restrictions. These included increased use of simulation labs to improve procedural skills, creation of a structured intern curriculum, and extension of the internal medicine residency program (List 2).

List 2. UAB internal medicine resident suggestions regarding improvement of graduate medical education following implementation of the 2011 ACGME duty hour standards.

1. Extend internal medicine residency training programs beyond three years.

2. Improve and adapt current graduate medical education curriculum to better reflect the changing educational environment, including incorporation of board review questions into conferences and reduction of research-based conferences.

3. Development of a structured intern (PGY-2) curriculum to ensure key concepts and skills are mastered.

4. Increase use of simulation laboratories to improve procedural skills and clinical reasoning.

5. Remove logistical barriers to increased time dedicated to learning, such as documentation requirements for interns.

6. Encourage proactive, extracurricular learning through the use of online resources.

## Discussion

Our study is one of the first reports to gauge the impact of the new 2011 ACGME duty hour standards among internal medicine residents. Resident perceptions regarding the impact of the July 2011 ACGME work-hour restrictions on graduate medical education were reported to affect learning in many ways. Most residents noted a decline in teaching by both faculty and upper level residents, and were concerned with the decrease in “hands-on” clinical education due to spending fewer hours in the hospital. These concerns extended to both procedural, as well as clinical reasoning skills that they perceived could only be honed via hands-on patient care.

Our findings validate published faculty concerns following the ACGME’s announcement of work hour limitations for residents and echo the few studies reporting on changes in graduate medical education outcomes following implementation of the 2003 ACGME duty hour standards [3,14,16,18,19]. More recently, a study assessing perceptions and attitudes of surgical residents via electronic survey found that 75% of responding residents expressed dissatisfaction with the 2011 duty hour standards and a large majority (75% of PGY-1 and 94% of PGY-2 through PGY-5) expressed concerns about the adverse impact of the work hour restrictions on the education of interns [20].

While all residency classes reported many of the same concerns regarding the impact of the work hour restrictions on graduate medical education, their opinions varied. The most notable divergence was regarding the preparedness of interns for their role as upper level residents. These diverging opinions on competency of skill set, as well as the shift in resident roles and responsibilities that have accompanied the changes in duty hour standards, have led to a reported disruption in team cohesiveness that may negatively impact the sharing of medical knowledge among these groups. Whether this impact on team dynamics will diminish as all residents are trained under the same duty hour standards, or whether it is an early indicator of a schism that will persist between residents of different training levels is yet to be seen.

Positive changes were also reported after the implementation of the 2011 ACGME duty hour standards. Both decreased intern fatigue and the increased availability of independent study time for PGY-1 residents were highlighted. Such positives were also reported in the literature following the 2003 ACGME duty hour standards [7,8,13,17,21]. Whether these improvements in resident quality of life result in enhanced patient safety and better clinical outcomes is still being determined and the impact of these changes on resident education remain underexplored. A more recent study assessing sleep patterns among pediatric residents in a control group (every fourth night, 30-hour call) versus an intervention group adhering to the 2011 guidelines suggested that despite being better rested, residents in the intervention arm were often more stressed and reported a poorer educational experience compared to their control group counterparts due to the compression of their workload into a shorter day [22]. Our residents generally reported feeling as though being more rested improved their ability to learn both inside and outside of the hospital; however, the long-term impact of fewer hours in the hospital on educational experience remains unknown. Though the impact of work hour changes on patient safety and clinical outcomes are not yet clear, questions surrounding the impact of these changes on our graduate medical education process need to be explored concomitantly, as poorly trained residents would certainly negatively impact patient outcomes.

Our study has limitations. It was conducted at a single academic medical institution with a small number of residents (n = 34) from only one medical specialty. For these reasons, our results may not be generalizable to other sites or specialties. However, our study is one of the earliest to address the educational impact of the 2011 ACGME duty hour standards in the cognitively oriented field of Internal Medicine. Additionally, though our sample size was small, our study involved residents at all levels of training and utilized focus group methodology to gain a clear understanding of resident perceptions unobtainable through standard survey methodology. Although focus groups were conducted and data was reviewed until a saturation of themes was reached, it is possible that additional themes may have arisen with more focus group discussions. Finally, individuals self-selected to participate in our study and thus may be biased by individuals with strong negative or positive attitudes associated with the implementation of the 2011 ACGME duty hour standards.

More studies are needed to further elucidate the impact of the current duty hour standards on graduate medical education. A decline in residency training quality could negatively impact long term patient safety and clinical outcomes, potentially undermining the gains from work hour changes. The impact of work hour changes on resident education must be more closely observed and the development of methodologies to expand the learning environment and promote extracurricular learning may be important to supplement traditional graduate medical education in the rapidly changing training environment.

## Competing interests

The authors of this manuscript have no competing financial or non-financial interests to report.

## Authors’ contributions

CRN made contributions to conception and study design, acquisition and analysis of data, and in drafting/revising the manuscript. AC made contributions to study design, data analysis, and drafting/revising the manuscript. BR made contributions to study design, data analysis, and drafting/revising the manuscript. DDD made contributions to study design and drafting/revising the manuscript. JMR made contributions to conception and study design, drafting/revising the manuscript. MP made contributions to conception and study design, drafting/revising the manuscript. EDS made contributions to conception and study design, drafting/revising the manuscript. ALG made contributions to conception and study design, drafting/revising the manuscript. JB made contributions to conception and study design, drafting/revising the manuscript. JHW made contributions to conception and study design, data acquisition and analysis and drafting/revising the manuscript. All authors read and approved the final manuscript.

## Pre-publication history

The pre-publication history for this paper can be accessed here:

http://www.biomedcentral.com/1472-6920/14/84/prepub

## References

[B1] Professionalism ATFoQCa. The ACGME 2011 Duty Hour Standards Enhancing quality of care, supervision, and resident professional development 2011 http://www.acgme.org/acgmeweb/Portals/0/PDFs/jgme-monograph[1].pdf. Accessed March 15, 2012

[B2] Brunworth JD Sindwani R Impact of duty hour restrictions on otolaryngology training: divergent resident and faculty perspectives Laryngoscope 2006 116 7 1127 1130 10.1097/01.mlg.0000224348.44616.fb 16826046

[B3] Cohen-Gadol AA Piepgras DG Krishnamurthy S Fessler RD Resident duty hours reform: results of a national survey of the program directors and residents in neurosurgery training programs Neurosurgery 2005 56 2 398 403 discussion 398–403 10.1227/01.NEU.0000147999.64356.57 15670388

[B4] de Virgilio C Yaghoubian A Lewis RJ Stabile BE Putnam BA The 80-hour resident workweek does not adversely affect patient outcomes or resident education Curr Surg 2006 63 6 435 439 discussion 440 10.1016/j.cursur.2006.03.006 17084773

[B5] Espey E Ogburn T Puscheck E Impact of duty hour limitations on resident and student education in obstetrics and gynecology J Reprod Med 2007 52 5 345 348 17583230

[B6] Fitzgibbons SC Chen J Jagsi R Weinstein D Long-term follow-up on the educational impact of ACGME duty hour limits: a pre-post survey study Ann Surg 2012 256 6 1108 1112 10.1097/SLA.0b013e31825ffb33 23069864

[B7] Fletcher KE Reed DA Arora VM Patient safety, resident education and resident well-being following implementation of the 2003 ACGME duty hour rules J Gen Intern Med 2011 26 8 907 919 10.1007/s11606-011-1657-1 21369772 PMC3138977

[B8] Goitein L Shanafelt TD Wipf JE Slatore CG Back AL The effects of work-hour limitations on resident well-being, patient care, and education in an internal medicine residency program Arch Intern Med 2005 165 22 2601 2606 10.1001/archinte.165.22.2601 16344417

[B9] Jagannathan J Vates GE Pouratian N Sheehan JP Patrie J Grady MS Jane JA Impact of the accreditation council for graduate medical education work-hour regulations on neurosurgical resident education and productivity J Neurosurg 2009 110 5 820 827 10.3171/2009.2.JNS081446 19409028

[B10] Jagsi R Shapiro J Weissman JS Dorer DJ Weinstein DF The educational impact of ACGME limits on resident and fellow duty hours: a pre-post survey study Acad Med 2006 81 12 1059 1068 10.1097/01.ACM.0000246685.96372.5e 17122470

[B11] Karamanoukian RL Ku JK DeLaRosa J Karamanoukian HL Evans GR The effects of restricted work hours on clinical training Am Surg 2006 72 1 19 21 16494176 10.1177/000313480607200105

[B12] Lin GA Beck DC Garbutt JM Residents’ perceptions of the effects of work hour limitations at a large teaching hospital Acad Med 2006 81 1 63 67 10.1097/00001888-200601000-00017 16377823

[B13] Lin GA Beck DC Stewart AL Garbutt JM Resident perceptions of the impact of work hour limitations J Gen Intern Med 2007 22 7 969 975 10.1007/s11606-007-0223-3 17468888 PMC2219723

[B14] Mir HR Cannada LK Murray JN Black KP Wolf JM Orthopaedic resident and program director opinions of resident duty hours: a national survey J Bone Joint Surg Am 2011 93 23 e1421 e1429 22159864 10.2106/JBJS.K.00700

[B15] Peabody T The effect of work hour restrictions on the education of orthopaedic surgery residents Clin Orthop Relat Res 2006 449 128 133 16735877 10.1097/01.blo.0000224037.54345.77

[B16] Vaughn DM Stout CL McCampbell BL Groves JR Richardson AI Thompson WK Dalton ML Nakayama DK Three-year results of mandated work hour restrictions: attending and resident perspectives and effects in a community hospital Am Surg 2008 74 6 542 546 discussion 546–547 18556998

[B17] Fletcher KE Underwood W 3rd Davis SQ Mangrulkar RS McMahon LF Jr Saint S Effects of work hour reduction on residents’ lives: a systematic review JAMA 2005 294 9 1088 1100 10.1001/jama.294.9.1088 16145030

[B18] Choby B Passmore C Faculty perceptions of the ACGME resident duty hour regulations in family medicine Fam Med 2007 39 6 392 398 17549647

[B19] Nuthalapaty FS Carver AR Nuthalapaty ES Ramsey PS The perceived impact of duty hour restrictions on the residency environment: a survey of residency program directors Am J Obstet Gynecol 2006 194 6 1556 1562 10.1016/j.ajog.2005.10.215 16731071

[B20] Lee DY Myers EA Rehmani SS Wexelman BA Ross RE Belsley SS McGinty JJ Bhora FY Surgical residents’ perception of the 16-hour work day restriction: concern for negative impact on resident education and patient care J Am Coll Surg 2012 215 6 868 877 10.1016/j.jamcollsurg.2012.08.005 23040454

[B21] Zahrai A Chahal J Stojimirovic D Schemitsch EH Yee A Kraemer W Quality of life and educational benefit among orthopedic surgery residents: a prospective, multicentre comparison of the night float and the standard call systems Can J Surg 2011 54 1 25 32 10.1503/cjs.050809 21251429 PMC3038360

[B22] Gordon MB Sectish TC Elliott MN Klein D Landrigan CP Bogart LM Amrock S Burke A Chiang VW Shuster MA Pediatric residents’ perspectives on reducing work hours and lengthening residency: a national survey Pediatrics 2012 130 1 99 107 10.1542/peds.2011-3498 22665414

